# Small Schiff Base Molecules—A Possible Strategy to Combat Biofilm-Related Infections

**DOI:** 10.3390/antibiotics13010075

**Published:** 2024-01-12

**Authors:** Maria Coandă, Carmen Limban, Diana Camelia Nuță

**Affiliations:** Department of Pharmaceutical Chemistry, Faculty of Pharmacy, Carol Davila University of Medicine and Pharmacy, 6 Traian Vuia Str., 020950 Bucharest, Romania; maria.coanda@drd.umfcd.ro (M.C.); diana.nuta@umfcd.ro (D.C.N.)

**Keywords:** Schiff base, antibiofilm, antimicrobial, imines, oximes, hydrazones

## Abstract

Microorganisms participating in the development of biofilms exhibit heightened resistance to antibiotic treatment, therefore infections involving biofilms have become a problem in recent years as they are more difficult to treat. Consequently, research efforts are directed towards identifying novel molecules that not only possess antimicrobial properties but also demonstrate efficacy against biofilms. While numerous investigations have focused on antimicrobial capabilities of Schiff bases, their potential as antibiofilm agents remains largely unexplored. Thus, the objective of this article is to present a comprehensive overview of the existing scientific literature pertaining to small molecules categorized as Schiff bases with antibiofilm properties. The survey involved querying four databases (Web of Science, ScienceDirect, Scopus and Reaxys). Relevant articles published in the last 10 years were selected and categorized based on the molecular structure into two groups: classical Schiff bases and oximes and hydrazones. Despite the majority of studies indicating a moderate antibiofilm potential of Schiff bases, certain compounds exhibited a noteworthy effect, underscoring the significance of considering this type of molecular modeling when seeking to develop new molecules with antibiofilm effects.

## 1. Introduction

Clinically relevant microbial biofilms are defined as “aggregated microbial cells surrounded by a polymeric self-produced matrix, which may contain host components”, suspended or attached to a surface [1]. Biofilm-related infections attracted the attention of scientists 50 years ago in the context of cystic fibrosis, and their impact on the medical field has grown ever since [2]. These infections may be tissue-related (chronic otitis media, chronic sinusitis, chronic laryngitis, dental plaque, endocarditis, cystic fibrosis, kidney stones, biliary tract infections, urinary tract infections, osteomyelitis, wound infections, etc.) or associated with medical devices (contact lenses, endotracheal tubes, cardiac devices or catheters) [1,3]. Some examples of biofilm forming pathogens are: *Pseudomonas aeruginosa*, *Staphylococcus aureus*, *Haemophilus influenzae*, *Staphylococcus epidermidis*, *Streptococci*, *Enterococci* and *Candida* spp. [4].

Biofilm formation requires four stages: (i) attachment of the mobile microorganism to a surface, (ii) colonization, (iii) development and maturation of biofilm and (iv) dispersion and propagation [5,6]. Attachment is mediated by cilli, flagella, surface proteins of microorganisms and rugosity of the surface [7]. It is reversible at first and then becomes irreversible, triggering transcription of specific genes for signalling molecules and extracellular polymeric substances (EPS). Colonization involves growth and division processes and EPS synthesis [5]. A mature biofilm consists of three layers: the biofilm nucleus, membranes of basal microorganisms and external mobile planktonic cells. It is a complex mixture of water, microbial cells, proteins, aminoacids and polysaccharides [6]. Dispersion is mediated by external factors or by self-digestion and contributes to dissemination of infection [7]. There are two important features of biofilm (sessile) growth compared with the free-floating (planktonic) state that contributes to pathogenicity: increased tolerance to antibiotic treatment and persistence in the host, despite inflammation and immune response [1]. The major consequence is that biofilm infections are hard to treat and usually become chronic [8].

Antibiotherapy is active on planktonic microbial cells, but its effectiveness against sessile states is variable, as established biofilms are usually recalcitrant to conventional antibiotics [9]. Treatment may require higher doses of antimicrobials, prolonged duration [8,10], combination therapy [11,12] or special modes of administration (nebulized antibiotics) [13]. 

**Figure 1:**
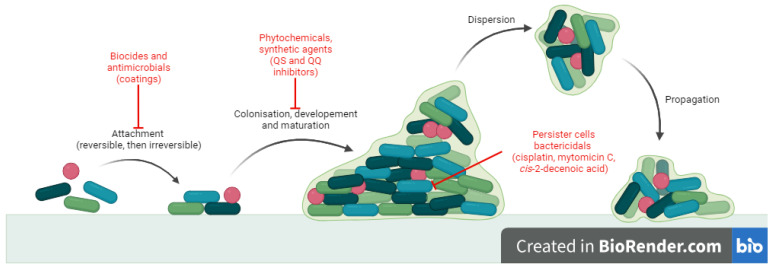
Antibiofilm mechanisms of action for small molecules.

There is a constant need to develop alternative antibiofilm strategies, and extensive research has been conducted in this direction [10]. Antibiofilm small molecules are relevant because they target stages in biofilm development which are different to those of normal planktonic state [9]. The mechanisms (Figure 1) may involve: blocking microbial adhesion (biocides [14], antibiotics [15] and impregnated coatings [16]), inhibition of microbial communication (quorum sensing inhibitors and quorum quenching) [17] and killing cells inside the biofilm (persisters or non-growing cells) (cisplatin, *cis*-2-decenoic acid, colistin, mytomicin C) [18,19,20,21]. In particular, strategies like quorum sensing inhibition may prove useful because they do not necessarily affect bacterial growth but they reduce virulence, thus increasing susceptibility of microorganisms to antibiotics and to host immune cells without the risk of antibiotic resistant [17]. 

Schiff bases are compounds with the structure R’N=CR_2_ (R’ ≠ H) [22], traditionally formed in the reaction of alkyl/aryl aldehydes or ketones with primary amines [23]. Many considered them to be synonymous with azomethines (RN=CR_2_, R ≠ H), both being a particular case of imines (RN=CR_2_, R = H, hydrocarbonyl) [22]. They are all part of carbonyl compound derivatives, formed in the reaction with basic nucleophiles (amines and their derivatives—hydroxylamine, hydrazine, N-acyl-hydrazine and semicarbazide). Therefore, this class of compounds also includes oximes (RR’C=NOH), hydrazones (R_1_R_2_C=N-NH_2_), N-acyl-hydrazones (R_1_R_2_C=N-NH-CO-R) and semicarbazones (R_1_R_2_C=NNH-(CO)-NH_2_) [14].

Schiff bases have numerous applications including coordination chemistry [23], catalysis [24], chemosensors [25] and intermediates in synthesis [26,27]. They also exhibit a variety of biological applications: antibacterial [28,29,30,31,32,33,34], antifungal [34,35,36], antiviral [37,38], antimalarial [39,40], antituberculosis [41,42,43], anthelmintic [44,45], urease inhibitors [46,47,48], anticancer [49,50,51], antidyslipidemic [52], antidiabetic [53,54], antidepressant [55,56,57], anticonvulsant [58,59], neurodegenerative disorder treatment [60,61], anti-inflammatory [62] and antioxidant [53,54].

**Figure 2:**
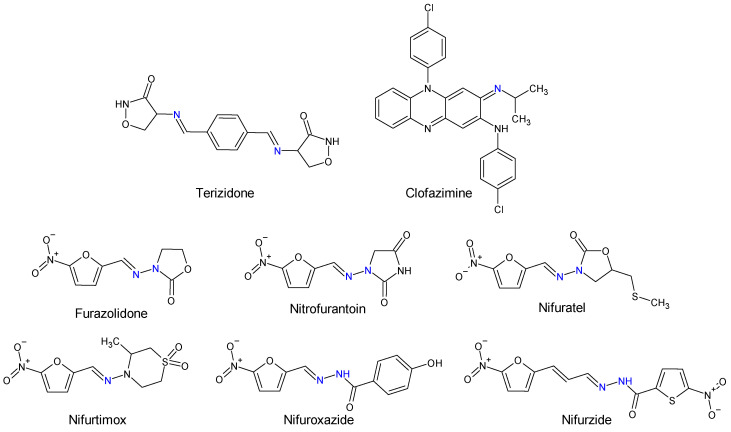
Structure of Schiff base and *N*-acyl-hydrazone medicines.

Human and veterinary therapy benefits from several antibacterial drugs recognized as Schiff bases. In multidrug-resistant tuberculosis (MDR-TB), a longer treatment regimen includes two Schiff bases which act on *Mycobacterium tuberculosis* cell wall: bacteriostatic terizidone (Figure 2), a cycloserine derivative, analogue of D-alanine and anti-leprosy clofazimine, which is an iminophenazine [63,64].

**Figure 3:**
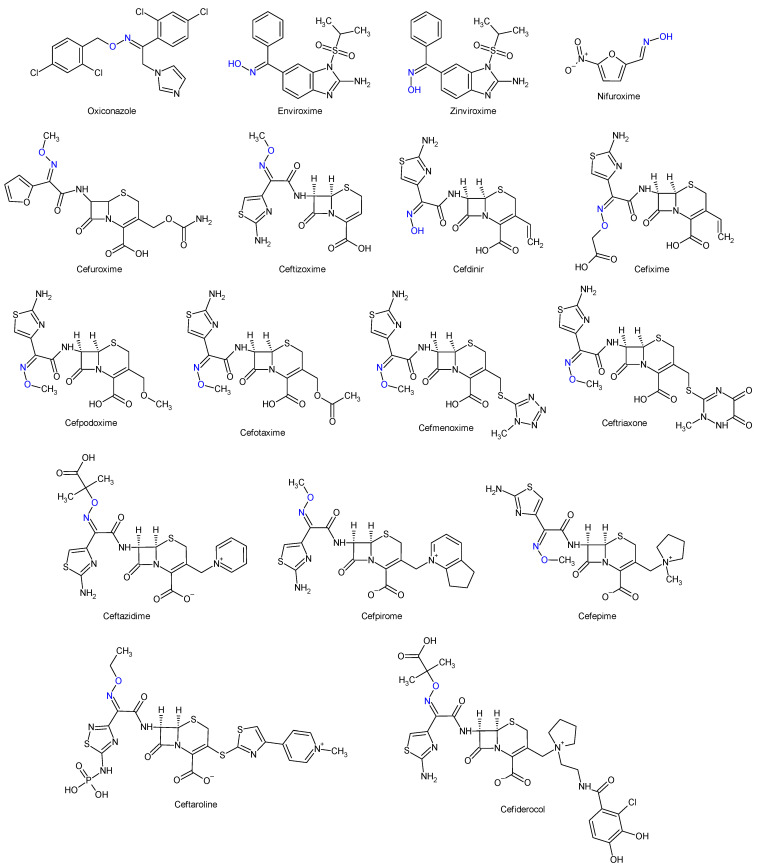
Structure of oxime medicines.

Oximes and hydrazones are moieties frequently used in medicinal chemistry. Examples of oxime drugs utilized in antimicrobial therapy include: cephalosporins (second-generation—cefuroxime, third-generation—cefdinir, cefixime, cefpodoxime, ceftazidime, cefmenoxime, ceftizoxime, ceftriaxone, cefotaxime, cefpirome, fourth-generation—cefepime, fifth-generation—ceftaroline, cefiderocol) [65,66,67,68], as well as antifungal (oxiconazole) [69], antiviral (enviroxime, zinviroxime) [70,71] and anti-infective (nifuroxime) medications [72] (Figure 2 and Figure 3).

N-acyl-hydrazones derivatives of 5-nitrofuran are prodrugs which act against different types of pathogens [73]. Some examples are nifuratel (antibacterial, antifungal, antitrichomonal agent) [74], furazolidone (antiprotozoal agent, gynaecological antiinfective and antiseptic) [75,76], nifurzide, nifuroxazide (intestinal antiinfectives, antidiarrheal agents) [77], nitrofurantoin (antibacterial) [78] and nifurtimox (antitrypanosomal and antileshmanial agent) [79]. Along with their stated antiinfective indications, studies have explored other possible applications of these drugs. Nifuratel—activity against *Leishmania* spp. [80,81], nifuroxazide—quorum sensing and biofilm inhibition [82], antischistosomal activity [83]. *N*-acyl-hydrazones 5-nitrofurans also exhibit anticancer properties, inhibiting different pathways in cancer cell cycles: signal transducer and activator of transcription 3 (STAT3) (nifuroxazide [84], nifuratel [85]), aldehyde dehydrogenase 1 (ALDH1) (nifuroxazide [86]) and nuclear factor kappa B signalling (furazolidone [87]) (Figure 2 and Figure 3). 

Numerous reviews have explored the antimicrobial potential of Schiff bases [88,89,90], as well as Schiff base-derived metal complexes [89,91,92,93], nanoparticles and modified chitosan [90,94]. While there are reports on the antibiofilm potential of Schiff base metal complexes [95,96], there is limited information on small molecules. 

Therefore, this review aims to provide a comprehensive overview of the existing scientific literature on small molecules classified as Schiff bases with antibiofilm properties.

## 2. Results

The literature survey is summarized in Table 1. The relevant articles were selected and divided into two categories based on structure: classical Schiff bases and oximes and hydrazones. 

## 3. Discussion

Due to their ease of synthesis and their wide range of applications, salicylaldehyde Schiff bases are frequently cited in the relevant literature. These compounds demonstrate antimicrobial potential, both as simple ligands and as metal complexes [56,57,58]. The antimicrobial activity is directly influenced by substitutions on the salicyl moiety, with halogenation exerting a noticeable impact in particular [58].

Taurine-5-bromosalicylaldehyde Schiff base (TBSSB) is a potassium salt of 2-{[1-(5-bromo-2-hydroxyphenyl)-meth-(*Z*)-ylidene]-amino} ethanesulfonic acid, with antistaphylococcal [97] and antimycobacterial potential [98] that is active against both planktonic and sessile forms. TBSSB was bactericidal against *S. aureus* (MIC 32 μg/mL), affecting membrane integrity and also preventing biofilm formation at 8 μg/mL [97]. The sulfonic acid group seems essential to antistaphylococcal activity [97]. The antimycobacterial effect was even better. TBSSB completely inhibited *M. smegmatis* mc^2^155 growth at > 60 μg/mL, presenting greater cell wall destruction compared with *S. aureus* alongside alterations in cell division. Additionally, it exhibited dose-dependent inhibition of *Mycobacterium* biofilm formation [31].

*p*-Aminobenzoic acid (PABA) is an amino acid derivative, implicated in folate biosynthesis in microbial cells [133]. Due to its importance for bacterial viability, it serves as a target for antimicrobial therapy [134]. Therefore, obtaining hybrid molecules is a direction of molecular development [135] in the search for new anti-infective agents.

Starting from a series of Schiff base derivatives of *p*-aminobenzoic acid and halogenated salicylaldehydes (compound **1**), me-too analogues were synthesized and tested for antimicrobial, antibiofilm and cytotoxicity activities [99]. The design approaches were as follows: isomerization (*m*-aminobenzoic acid (MABA) derivatives **2**) esterification (methyl esters **3**, and ethyl esters **4**) amide formation (N-phenylamides **5**) duplication of azomethine bond (3,5-diaminobenzoic acid (DABA) derivatives **6**). The Schiff bases obtained were active against Gram-positive strains, having MIC from 7.81 μM. The corresponding amines presented no antimicrobial effect. Diiodo derivatives (**2b**, **3b** and **6b**) were comparable in action to bacitracin (SA: MIC 7.81 μM, EF: 15.62 μM). No activity was observed against *Mycobacterium* strains. Regarding antifungal activity, the analogues surpassed the original PABA Schiff bases. Derivatives **2**, **5** and **6** exhibited broad-spectrum activity, *C. albicans* and *T. interdigitale* being the most susceptible. The best results were obtained for diiodo analogues (**2b**, **5b**, **6b**), having MICs comparable to fluconazole (CA: 6.5 μM). The antibiofilm evaluation was performed on two strong biofilm producers: methicillin-resistant *S. aureus* ATCC 43300 and *S. epidermidis* ATCC 1228. Compound **3b** (methyl (*E*)-4-[(2-hydroxy-3,5-diiodobenzylidene)amino]benzoate) was only moderately active (MRSA: MBIC 781.25–1562.5 μg/mL, MBEC 1562.5–3125.0 μg/mL; SE: MBIC 781.25–1562.5 μg/mL, MBEC > 1562.5 μg/mL) compared with ciprofloxacin (MRSA: MBIC 0.381 μM, MBEC 48.8 μg/mL, SE: MBIC 0.381–0.7625 μg/mL, MBEC 97.6–195.3 μg/mL). The methyl ester was also the least cytotoxic. Thus, 3,5-dihalogenosalicylic scaffold is essential for antimicrobial activity—iodine atoms preferred (3,5-diiodo, followed by 3-iod-5-chloro- substitution) [99].

Simplifying the structure of rafoxanide (a veterinary anthelmintic) by changing the amide group with azomethine and eliminating the phenoxy substituent, an imine analogue, (*E*)-2-{[(4-chlorobenzyl)imino]methyl}-4,6-diiodophenol (**7**) was obtained [100]. Compound **7** presented selectivity on Gram-positive bacteria, exhibiting antistaphylococcal (MIC 15.625–62.5 μM) and antienterococcal (MIC 62.5–125 μM) activities on reference strains and clinical isolates. The action is bactericidal, and the mechanism indicated inhibition of protein synthesis pathways followed by inhibition of nucleic acid and peptidoglycan production. It exhibits moderate-to-good antibiofilm activity against MRSA and SE (MRSA: MBIC 62.216–124.432 μg/mL, MBEC 124.432–248.863 μg/mL; SE: MBIC 31.108–62.216 μg/mL, MBEC 124.432–248.863 μg/mL) compared with ciprofloxacin (MRSA: MBIC 0.381 μM, MBEC 48.8 μg/mL, SE: MBIC 0.381–0.763 μg/mL, MBEC 97.6–195.3 μg/mL). Due to its bactericidal action, compound **7** seemed to reduce bacterial metabolic activity and inhibit the viability of the released planktonic cells from the biofilm [100].

Combining two pharmacophores—salicylaldehyde and **s**ulphonamides—two series of Schiff base analogues of sulfamethoxazole (compounds **8**), sulfathiazole (compounds **9**) and sulfamethazine (compound **10**) were synthesized [101]. The influence of the substitution of salicylaldehyde moiety (R^2^) on antimicrobial activity was investigated. Gram-positive bacteria, especially *Staphylococci*, were susceptible to the action of the analogues (MIC ≥ 15.62 μM), including clinical isolates (MIC ≥ 3.91 μM) and resistant species (methicillin-resistant *S. aureus*, MRSA, cotrimoxazole resistant species). Interestingly, the Schiff bases were bactericidal in action compared with sulfonamides and active against cotrimoxazole resistant bacteria, exhibiting no cross-resistance. Eight compounds (**8c**–**d**, **9b**–**d**, **10a**–**c**) had MICs (15.62–31.25 μM) comparable to bacitracin (MIC 7.81-15.62 μM) against *S. aureus*. Once more, the most favourable outcome was achieved with the 3,5-dihalogen substitution on the salicylaldehyde molecule, particularly with the presence of at least one iodine atom, making sulfamethazine derivatives (**10**) the most potent [101].

4-[(3,5-Dichloro-2-hydroxybenzylidene)amino]-N-(4,6-dimethylpyrimidin-2-yl)benzene-sulfonamide (**10a**) inhibited MRSA and *S. epidermidis* biofilm formation (MBIC 390.6–781.25 μM, MBEC > 3462 μM) being inferior to ciprofloxacin (MRSA: MBIC 0.381 μg/mL, MBEC 48.8 μg/mL, SE: MBIC 0.381–0.763 μg/mL, MBEC 97.6–195.3 μg/mL). The compound was not able to disrupt the preformed matrix [101]. 

5-(4-Methylpiperazin-1-ylsulfonyl)benzylidene)anilines (**11a**–**f**) were synthesized and evaluated for antibacterial and anti-*Candida* actions [102]. The antibacterial activity varied among the strains and was influenced by the radical R used. *B. subtilis* was the most susceptible, followed by *P. aeruginosa*. Unsubstituted **11a** was more potent than the reference (ciprofloxacin—MIC 50 μg/mL) against PA, with electron-donating groups (4-OCH_3_, **11f**) increasing the activity. For *S. aureus* and *E. coli* biofilm inhibition, the compounds were inferior to ciprofloxacin. The most favourable substituent was the electron-withdrawing CF_3_ in *ortho* or in *meta* position (**11b**, **11c**). Electron-donating OH seemed essential for antifungal and antibiofilm activity. Derivatives **11d** (2-OH), **11c** (3-CF_3_) and **11e** (4-OH) surpassed fluconazole (MIC 50.0 μg/mL) in terms of anti-*Candida* activity. A similar trend was observed for fungal antibiofilm action, with compound **11d** (2-OH) being the most active, followed by **11a** (H), **11b** (3-CF_3_) and **11e** (4-OH) (fluconazole, IC_50_ 40 μM). Compound **11d** (2-(2-Ethoxy-5-(4-methylpiperazin-1-ylsulfonyl)benzylideneamino)phenol) inhibited the formation of *C. albicans* biofilm without affecting planktonic cells, which may indicate a quorum sensing mediated mechanism of action. The docking study against *Candida* secreted aspartyl protease (SAP5), the enzyme responsible for cell-to-cell adhesion and biofilm formation [136], indicated that the **11d** is held in place by van der Waals interactions, while 4-methylpiperazine ring form hydrophobic interactions with amino acids at the active site. The azomethine group is also responsible for strong van der Waals hydrophobic and charge bonds interactions with important active site amino acid residues (Ile12, Lys83, Gly85, Asp86, Gly220, Thr221, Thr222, Thr222, Ile223, Tyr225 and Ile305) [102]. 

4-(*o*-Methoxyphenyl)-2-aminothiazole was reported to possess antibacterial and antibiofilm potential [137]. Its Schiff bases with substituted salicylaldehydes (**12a**–**f**) and 2-hydroxy-1-naphtylaldehyde (**12g**) were synthesized and evaluated for the same effects [103].

4-Bromo-2-(((4-(2-methoxyphenyl)thiazol-2-yl)imino)methyl)phenol (**12f**) and 2-(((4-(2-methoxyphenyl)thiazol-2-yl)imino)methyl)naphthalen-1-ol (**12g**) exhibited antibacterial action against *B. subtilis* (MIC 25 μg/mL). Compound **12g** was also active against *E. coli* (MIC 100 μg/mL) [103], with Schiff bases surpassing the parent amine (amine: MIC 250 μg/mL for *B. subtilis*, 500 μg/mL for *E. coli*) [137]. Regarding antibiofilm potential, compounds **12f** and **12g** were able to inhibit *P. aeruginosa* biofilm formation but they do not affect the viability of the cell, suggesting a quorum sensing mechanism of action [103]. 

A series of Schiff bases starting from 2-amino-5-chloro-benzophenone was obtained using microwave irradiation and evaluated for antibiofilm and antibacterial activity [104]. Twelve compounds presented MBIC under 100 μg/mL (**13a**–**k**). The antibacterial/ antibiofilm activity depended on the type and nature of substituents (R, R^1^), with electron-donating groups (methoxy, hydroxy) and halogens being favourable. The salicylaldehyde derivative (**13d**) was only active against *S. mutans*. The introduction of halogen atoms extended the action to *S. aureus* (F—**13e**), *K. pneumoniae* (Br—**13f**) and *P. mirabilis* (Cl, Br—**13g**). The acridine derivative (**13l**) and compounds **13a–c** inhibited both Gram-positive and Gram-negative bacteria, being inferior to cefixime (MIC 41 μg/mL). *S. aureus* biofilm was significantly disrupted by compounds **13i**, **13k** and **13g**, while **13i** was also active against preformed biofilm of *P. mirabilis* [104]. 

4-aminophenazone Schiff bases with different substituted cinnamaldehydes (**14a**–**c**) were obtained and tested for antimicrobial and antibiofilm activity. 4-(2-Bromo-3-phenyl-2-propenylideneamino)-1,5-dimethyl-2-phenylpyrazol-3-one (**14a**) exhibited broad antimicrobial spectrum. It inhibited all fungal strains and all tested bacteria, except *P. aeruginosa*, exhibiting bactericidal (*K. ozaenae*, *S. enterica*) or bacteriostatic effect (*E. gergoviae*). It reduced up to 90.41% of the biofilm of *C. tropicalis* and between 75–83% of *E. faecalis* and *S. aureus*. Compounds **14b** and **14c** were also active on biofilm [105].

Bacterial fatty acid synthetase may serve as the target for the development of new antibacterial agents. Triclosan and other 2-hydroxydiphenyl ethers demonstrated inhibition against enoyl-acyl carrier protein reductase (FabI), a key enzyme in fatty acid production [138,139]. Schiff bases and hydrazones have also been reported as inhibitors of staphylococcal β-ketoacyl carrier proteinsynthase III (encoded by FabH gene) [140,141]. In Gram-negative bacteria, PqsD—an enzyme implicated in *Pseudomonas* autoinductors synthesis—is structurally related to FabH. Thus, inhibitors of fatty acid synthetases may also act against PqsD [142].

**Figure 4:**
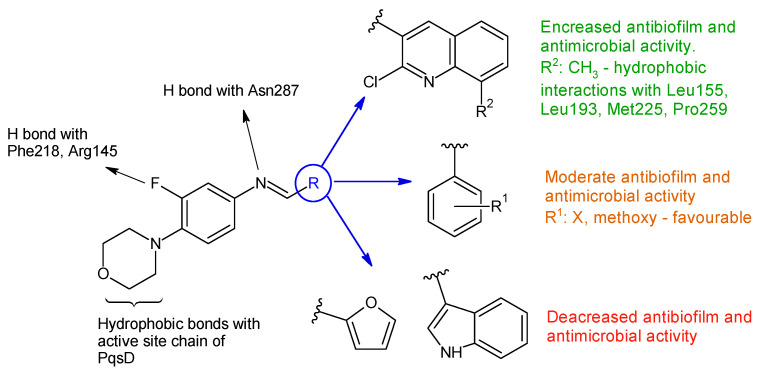
Structure–activity relationship for compounds **15**, including interactions with PqsD enzyme.

Linezolid derived Schiff bases were synthesized starting from 4-(4-amino-2-fluorophenyl)-morpholine in order to obtain PqsD enzyme inhibitors [106]. Biofilm inhibition varied according to radical R used. The quinoline derivatives (N-((2-chloroquinolin-3-yl)methylene)-3-fluoro-4-morpholinoaniline—**15h**, N-((2-chloro-8-methylquinolin-3-yl)methylene)-3-fluoro-4-morpholinoaniline—**15i**) exhibited the greatest activity against *P. aeruginosa* biofilm (**15h**—IC_50_ 12.97 ± 0.33 μM,**15i**—IC_50_ 15.63 ± 0.20 μM), surpassing linezolid (IC_50_ 15.93 ± 0.18 μM). They also presented good anti-*Pseudomonas* activity, comparable to linezolid (MIC 2.5 μg/mL) and antimicrobial action against *E. coli* and *B. subtilis* (**15h**, **15i**: MIC 5–10 μg/mL, linezolid: MIC 2–3 μg/mL). The docking studies of **15h** and **15i** against PqsD enzyme revealed van der Waals interactions, hydrophobic bonds (methylene groups of morpholine, methyl group of quinoline) and hydrogen bonds (fluorine atom of linezolid moiety, azomethine group) with amino acid residues of the active site (Figure 4). The phenyl derivatives (**15a**–**e**) exhibited only moderate antibiofilm and antimicrobial activities, with electron-donating groups (halogen and methoxy) slightly increasing the effect. Indolyl (**15g**) and furanyl (**15f**) derivatives presented no enhancement (Figure 4) [106].

**Figure 5:**
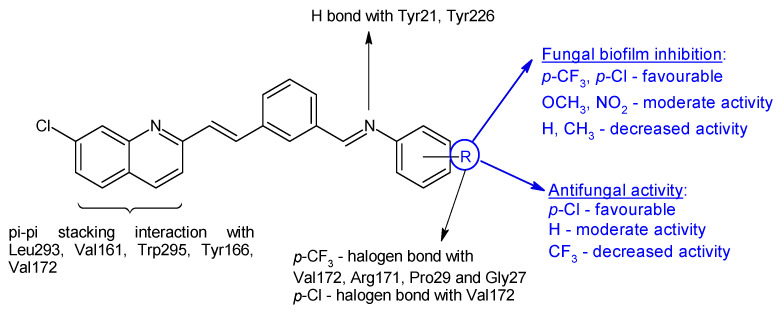
Structure–activity relationship for compounds **16**, including interactions with Als-3 adhesin of *C. albicans*.

*N*-(3-(-2-(7-chloroquinolin-2-yl)vinyl)benzylidene)anilines **16a**–**j** were synthesized based on 2-*n*-heptyl-4-hydroxyquinoline (HHQ) and 2-*n*-heptyl-3-hydroxy-4(1*H*)-quinoline (PQS- *Pseudomonas* Quinoline Signal) [107]. Radical R on the phenyl ring influenced antimicrobial and antibiofilm activities. Electronegative substituents improved antibacterial activity, with the *p*-chloro derivative **16b** being more potent than ciprofloxacin (MIC 50 μg/mL) on *E. coli*, while the *p*-trifluoromethyl derivative **16g** was the only one active on *P. aeruginosa* (MIC 91.5 μg/mL). *S. aureus* was most sensitive to *o*-trifluoromethyl derivative **16e** (MIC 55.3 μg/mL). For antifungal activity, the *p*-chloro substitution was beneficial, followed by unsubstituted derivative, while the *p*-trifluoromethyl derivative presented lower activity (**16f:** IC_50_ 174.4 μg/mL). Compounds with electronegative substituents such as trifluoromethyl (*N*-(3-(2-(7-Chloroquinolin-2-yl)vinyl)benzylidene)-4-(trifluoromethyl)aniline—**16g**, or chloro (*N*-(3-(2-(7-chloroquinolin-2-yl)vinyl)benzylidene)-4-chloroaniline—**16b**) on para position presented good fungal antibiofilm activity, comparable to fluconazole (IC_50_ 40 μM), whereas methoxy (**16h**) and nitro (**16i**, **16j**) derivatives indicated moderate activity (IC_50_ < 100 μM). The lack of substitution or hydrophobic groups (CH_3_) was unfavourable for biofilm inhibition (Figure 5). Docking studies against agglutinin-like protein (*C. albicans* Als-3 adhesin) indicated the formation of halogen bonds between para electronegative substituents and the active site, hydrogen bonds between imine group and Tyr21 and Tyr226 and π-π stacking interaction between naphthyl ring and Leu293, Val161, Trp295, Tyr166 and Val172 (Figure 5) [107].

*N*-phenyl-3-cyano-4-amino-pyrazole was used as the starting point for the design and development of antifungal Schiff bases. For both antifungal and antibiofilm activity, the optimal substituents were electron-withdrawing Br, NO_2_ and COOH. Compound **17i** (5-(4-bromobenzylideneamino)-1-(2,6-dichloro-4-(trifluoromethyl)phenyl)-1*H*-pyrazole-3-carbonitrile) was the most potent(MIC 42.6 μg/mL, IC_50_ 41.5 μM), and is comparable to fluconazole (MIC 50 μg/mL, IC_50_ 40 μM) [108].

*P. aeruginosa* uses two QS systems, *las* and *rhl*, that rely on transcriptional activators (LasR and RhlR, respectively) and autoinducer molecules (*N*-3-oxo-dodecanoyl-L-homoserine lactone, respectively *N*-butyryl-L-homoserine lactone) [143]. Interfering with these systems may serve as a strategy to reduce virulence and pathogenicity [144].

**Figure 6:**
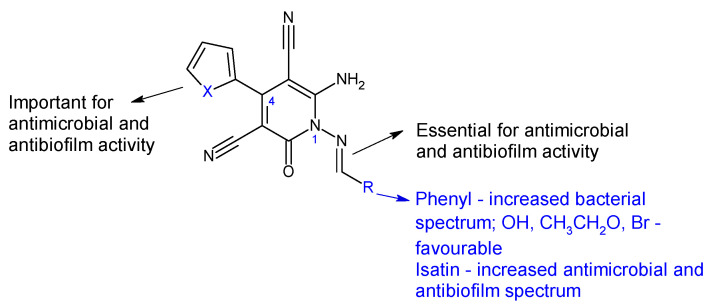
Structure–activity relationship for compounds **18, 19**.

Combining 6-amino-4-(thiophen-2-yl)-2-oxo-pyridine-3,5-dicarbonitrile (**18**) and 6-amino-4-(furan-2-yl)-2-oxo-pyridine-3,5-dicarbonitrile (**19**) with aromatic aldehydes and ketones, two series of antimicrobial Schiff bases (**18a**–**c** and **19a**–**c**) were synthesized under both conventional and green conditions (ceric ammonium nitrate catalysis) [109]. Biological screening revealed significant antimicrobial potential for azomethines, especially on Gram-negatives, surpassing or equalizing references (gentamicin and fluconazole) and biocidal modes of action. In terms of biofilm inhibition, azomethines were active against all tested strains, with MRSA and *E. coli* biofilms being the most susceptible. They were able to reduce *Las*R gene expression with 10–40% at 1/8 MIC compared with 60% for doxycycline. Compound **19a** presented extended antibacterial spectrum (*E. coli-* MIC 125 μg/mL, *K. pneumoniae-* MIC 15.6 μg/mL) comparable to gentamicin (MIC 250, 250 μg/mL). Compound **19b** was the most active against MRSA (MIC 62.5 μg/mL, gentamicin—MIC 125 μg/mL), with 5-bromo substituent on the phenyl ring being essential. Compound **18c** exhibited antifungal properties, surpassing fluconazole (MIC 62.5 μg/mL) and other derivatives (MIC 250 μg/mL), however, its effect on fungal biofilm was reduced (15.15%). Derivative **19c** (6-Amino-1-((1,3-dioxo-1,3-dihydro-2*H*-inden-2-ylidene)amino)-4-(furan-2-yl)-2-oxo-1,2-dihydropyridine-3,5-dicarbonitrile) was active against *P. aeruginosa* biofilm in both planktonic and sessile forms (gentamicin—BI 29.4%). It presented the highest degree of *Las*R gene expression inhibition among tested compounds (40%), significantly reduced *C. albicans* biofilm (75.0%) and surpassed fluconazole (57.6%). Structure–activity relationships revealed that azomethine is important for activity, which varied according to the substituents in position one (imine groups) and four (furanyl, thiophenyl) on pyridine moiety (Figure 6). Benzylidene and 1,3-dioxo-1,3-dihydro-2*H*-inden-2-ylidene increased the antibacterial spectrum, with electron-donating groups (hydroxy, ethoxy, bromo) being beneficial for MRSA and *E. coli* biofilm inhibition [109].

**Figure 7:**
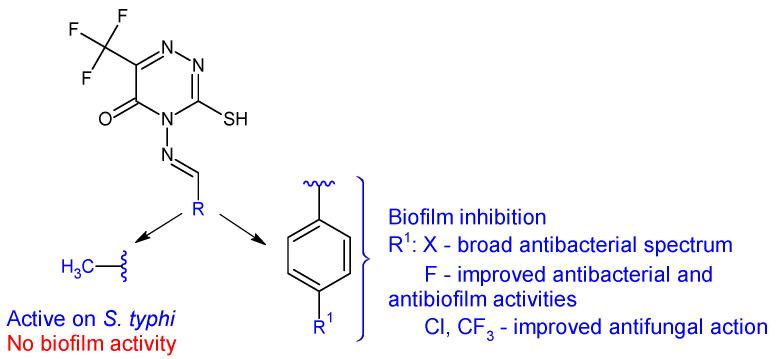
Structure–activity relationship for compounds **20**.

4-Amino-3-mercapto-6-(trifluoromethyl)-1,2,4-triazin-5(4*H*)-one was used as the starting point for the synthesis of six Schiff bases (**20a**–**f**) [110]. The in vitro biological evaluation revealed that Gram-positive and *S. typhi* were susceptible to all azomethines, while *E. coli* was tolerant to **20a** and **20e**. Halogen-substituted compounds **20b**–**d** exhibited broad spectrum antibacterial action, inhibiting in different percentages all tested strains (IR 4.57–87%). Compound **20b** (4-((4-fluorobenzylidene)amino)-3-mercapto-6-(trifluoromethyl)-1,2,4-triazin-5(4*H*)-one) was the most potent against *E. coli* and *S. aureus* (IR 87%, 75%; MIC 3.90 μg/mL) (ciprofloxacin—MIC 0.39 μg/mL). Compound **20a** (4-(ethylideneamino)-3-mercapto-6-(trifluoromethyl)-1,2,4-triazin-5(4*H*)-one) was most active against *S. typhi*, with a MIC of 7.81 μg/mL. Regarding antifungal assay, *A. flavus* was more sensitive to the action of Schiff bases than *A. niger*. Compound **20a** displayed good antifungal activity (IR up to 87%, MIC 15.62 μg/mL for *A. flavus*). Compounds **20c** and **20f** exhibited moderate fungal inhibition (IR 43–82%) but had better MICs than nystatin (3.90 μg/mL compared with 8.25 μg/mL for *A. flavus*). Phenyl derivatives **20b**–**f** inhibited the biofilm of *E. coli* and *S. aureus*, with **20b** being the most potent (IR 87.4%—*E. coli*, 72.4%—*S. aureus*) while **20a** was inactive. Hence, fluoro in para position of the benzene ring (**20b**) improves antibacterial and antibiofilm activities, whereas chloro and trifluoromethyl groups are beneficial for antifungal action (Figure 7) [110].

Isatins (1*H*-indole-2,3-diones) are synthetic and also naturally-occurring compounds, largely employed in organic synthesis due to their versatility and numerous applications [145]. 

A series of hybrid Schiff bases (**21a**–**f**) were synthesized by incorporating isatin, pyrazole and either piperidin-1ylsulfonyl or N-methylpiperazin-1ylsulfonyl into a single molecule [111]. These resultant molecules are amphiphilic in nature, stemming from the combination of polar groups (NH_2_, OH, SO_2_) with lipophilic hydrocarbon components, thereby enhancing their antibacterial potential. Compounds **21a**–**d** exhibited good antibacterial activity against tested strains (MIC 53.45–258.32 μM), comparable to norfloxacin (MIC 100.31–200.63 μM) and ciprofloxacin (MIC 48.33–96.68 μM). 5-Aminopyrazole moiety (R^2^: NH_2_) performed better than 5-hydroxypyrazole (R^1^: OH), with the most potent derivatives being **21b** and **21d**. Compounds **21b**–**f** exhibited good antifungal activity (MIC 106.91-208.59 μM), surpassing fluconazole (MIC 220.76 μM). Derivatives **21a**–**d** were fungicidal and bactericidal against all strains, except *S. aureus* (**21b**—bacteriostatic), *E. coli* (**21a**—bacteriostatic). Compound **21d** ((*E*)-3-({5-amino-1-benzoyl-4-[(*E*)-(4-hydroxyphenyl)diazinyl]-1*H*-pyrazol-3-yl}imino)-5-(piperidin-1-ylsulfonyl)indolin-2-one), the most active antimicrobial, inhibited the MRSA biofilm formation at concentrations of 0.007–0.03 mg/mL (BI 70.8 ± 2.3–89.9 ± 4.7%) [111].

In a subsequent study, Schiff bases and other imine derivatives were obtained condensing 5-((4-methyl-piperazin-1-yl)sulfonyl)indoline-2,3-dione with aminothiazole derivatives, sulfathiazole or thiourea [112]. Compounds **22a**–**c** exhibited good antibacterial activity (MIC 1.9–125 μg/mL), outperforming levofloxacin (MIC 8.1–130 μg/mL) with compound **22c** being the most active. Antifungal potential was reduced and compounds **22a** and **22d** presented moderate activity (MIC 62.5, 31.2 μg/mL), respectively (nystatin—MIC 3.9 μg/mL). Compounds **22a**–**d** were active against *S. aureus* biofilm (BI_50_ 1.95–15.6 μg/mL), with the sulfathiazole derivative **22b** (4-((5-((4-Methylpiperazin-1-yl)sulfonyl)-2-oxoindolin-3-ylidene)amino)-N-(thiazol-2-yl)benzenesulfonamide) being the most potent. Compounds **22c**, **22d** and **22e** were also active in this order against *P. aeruginosa* biofilms (BI_50_ 7.8 ± 0.13, 15.6 ± 0.32, 31.25 ± 0.051 μg/mL). Derivatives **22a**–**c**, especially **22c**, inhibited QS system of *E. coli*, known as fsr, thus presenting a QS mechanism of antibiofilm action [112].

Starting from two modified isatin molecules (1-(2-methylallyl)indoline-2,3-dione and 1-isobutylindoline-2,3-dione) and combining them with PABA, substituted *o*-aminobenzoic acids and *p*-aminomethylbenzoic acid, two series of Schiff base derivatives (**23**, **24**) were obtained and evaluated for antibacterial, antifungal and antibiofilm potential [113]. The hybrids inhibited Gram-positive bacteria. 2-Methylallyl derivatives (**23**) presented greater activity than isobutyl counterparts (**24**), with the best action being recorded for **23a** and **23b** (*S. aureus*, *B. subtilis*—MIC 0.09 mmol/L, 0.181 mmol/L) compared with imipenem (MIC 0.036 mmol/L). This difference may be attributed to the fact that 2-methylallyl is less hydrophobic than isobutyl and is able to penetrate membranes more easily and form π-H interactions with protein targets. Compounds **23a** ((*E*)-4-(1-(2-methylallyl)-2-oxoindolin-3-ylideneamino)benzoic acid) and **23b** ((*E*)-2-(1-(2-methylallyl)-2-oxoindolin-3-ylideneamino)-4-chlorobenzoic acid) showed over 55% biofilm inhibition against *S. aureus* and MRSA compared to 20% for chloramphenicol. The docking study against *B. subtilis* histidine kinase/Walk YycG, the enzyme involved in biofilm formation and bacterial virulence [146], revealed the importance of hydrogen bonds between Asp105 and **23a** for antibiofilm activity [113].

Schiff bases of methyl 12-aminooctadec-9-enoate (**25a**–**f**) were obtained and tested for antimicrobial and antibiofilm effects [114]. Gram-positive strains (*S. aureus*, *B. subtilis*) were susceptible to the action of the compounds, but Gram-negative bacteria remained resistant. The best antimicrobial effect was obtained for *p*-chloro derivative **25a**, followed by hydroxy and methoxy azomethines (**25c**, **25d**). All were inferior to ciprofloxacin (MIC 2.7 μM). N,N-dimethylamino and N,N-dimethyl cinnamyl Schiff bases presented moderate antibacterial activity (**25b**: MIC 17.6–35.2 μM, MBC 17.6–70.5 μM; **25e**: MIC 16.6–33.3 μM, MBC 16.6–66.6 μM). The mechanism seems bactericidal due to MBCs closed to MICs (MBC/MIC < 4) [147]. Regarding antibiofilm action, the order was maintained as **25a**, **25c**, **25d** and **25f** having IC_50_ under 10 μM (ciprofloxacin IC_50_ 0.99–1.53 μM) [114].

*N*-[(*E*)-4-bromo-2,5-diheptyloxybenzylideneamino]-2,4-dinitroaniline (**26**) was evaluated for antimicrobial, antibiofilm and antiquorum sensing activities at MIC and lower concentrations. *E. faecalis* and *Candida* strains were the most susceptible in terms of antimicrobial effect, while Gram-positive biofilms, especially of SA, were the most sensitive. It exhibited quorum sensing inhibition against *C. violaceum* and was able to reduce swarming motility of *P. aeruginosa* by 14.4–45.7% at MIC/4-MIC. Thus, it targets two steps in biofilm formation: communication and dispersion [115].

N-(2-hydroxybenzylidene)-2-hydroxypropanehydrazides (**27a**–**f**) and 2-hydroxy-N-((3-hydroxy-5-(hydroxymethyl)-2-methylpyridin-4-yl)-2-hydroxypropane-hydrazide (**27g**) were obtained by condensing (*S*)-lactic acid hydrazide with substituted salicylaldehydes or pyridoxal and tested for antibacterial and antibiofilm activities. The nitro derivative **27e** was active against *S. aureus*, while the pyridoxal derivative **27g** was able to inhibit *E. coli* (MIC 64 μg/mL for both derivatives and both strains). Also, they significantly reduced *P. aeruginosa* O1 biofilm formation at 1/16 and ¼ MIC [116].

2-Pyridinylhydrazone of substituted salicylaldehydes and pyridinylcarbaldehydes (**28a**–**f**) were active against *A. baumannii* planktonic and sessile cells. Electron-donating groups (R^1^: OH, OCH_3_) and nitro groups on salicylaldehydes moiety and 3 or 4-pyridinyl radical have a beneficial effect on antimicrobial and antibiofilm activities. All compounds were able to inhibit the biofilm of *A. baumannii* culture and clinical isolates (MIC < 200 μg/mL). Compounds **28a** ((*E*)-3-((2-(pyridin-2-yl)hydrazono)methyl)benzene-1,2-diol) and **28d** ((*E*)-4-nitro-2-((2-(pyridin-2-yl)hydrazono)methyl)phenol) exhibited the best antimicrobial activity (MIC 25 μg/mL) and acted as both biofilm inhibitors and disruptors (MIC < 25 μg/mL) [117]. 

Phenyl-2-(2-(1-phenylethylidene)hydrazinyl)thiazoles (**29**) were synthesized and evaluated against clinical isolates of *C*. *albicans* [118]. The thiazole ring was obtained in the reaction of thiosemicarbazones with substituted phenyl bromides. Biological assay revealed that compounds **29d**–**e** and **29a**–**c** were able to inhibit the biofilm formation at 50–100 μg/mL. Their action varied according to the substituents of the two benzene rings (R^1^, R^2^). Difluoro derivatives (**29d**–**e**) were equally potent, followed by methyl (**29c**), methoxy (**29b**) and unsubstituted (**29a**) analogues. Microscope imaging confirmed fungal biofilm formation reduction. Gene expression analysis indicated upregulation of inhibitory genes implicated in yeast-hyphae transition (bcy1, nrg1, tup1) and downregulation of genes responsible for *C*. *albicans* biofilm formation and virulence (als3, hwp1, ras1). The docking study indicated interactions between compounds and lanosterol 14-alpha-demethylase (van der Waals and hydrophobic bonds) [118].

**Figure 8:**
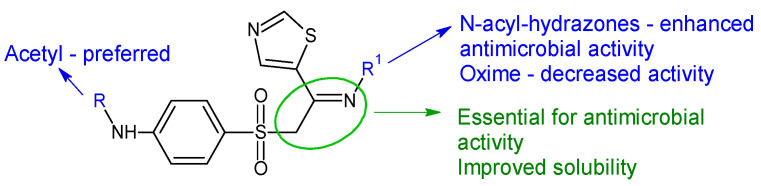
Structure–activity relationship for compounds **30**, **31**.

Starting from sulfathiazole, antistaphylococcal compounds were obtained by the isostere replacement of the nitrogen atom of a sulphonamide fragment by methylene carbonyl group, resulting in 2-(4-aminobenzene-1-sulfonyl)-1-(1,3-thiazol-5-yl)ethan-1-ones [148]. Through the continued optimization of these structures, the carbonyl fragment was changed to the imine group, resulting in oximes, hydrazones and N-acylhydrazones analogues [119]. The imine group was introduced in order to improve solubility and antimicrobial potential, while radical R and R^1^ modulated activity. Biological assay revealed that the ethyl imine group is beneficial for activity. Acetylated derivatives are preferred over simple amines. *N*-acyl-hydrazones were the most active against tested strains, with hydrazones being favourable and oximes decreasing the activity (Figure 8). Compound **31f** (*N*-(4-((2-(2-picolinoylhydrazono)-2-(thiazol-2-yl)ethyl)sulfonyl)phenyl)acetamide) was bactericidal against *E. faecalis*, standard and clinical isolates at MIC 1–4 μg/mL, surpassing norfloxacin (MIC 4–8 μg/mL) and sulfathiazole (MIC 128 μg/mL). It was also able to reduce the biofilm mass of *E. faecalis* by 35% at 6xMIC. Some possible mechanisms of action for compound **31f** are membrane damage, oxidative damage, inhibition of dihydrofolate synthetase and complexation of DNA [119]. 

(*E*)-*N’*-((5-nitrofuran-2-yl)methylene)quinoline-8-sulfonohydrazide (**32**) was obtained combining the pharmacophores 5-nitro-furan and quinoline and linked via a sulfonyl–hydrazone bond. Antimicrobial screening revealed antifungal potential against culture type fungal strains and clinical isolates (MIC 125–250 μg/mL) and modest antibacterial properties. The compound was able to inhibit *C. albicans* at 32.1 μg/mL, yeast-hyphae transition at 24.96 μg/mL and fungal biofilm formation (38% inhibition at MIC). Quinoline and furan rings form hydrophobic and aromatic interactions with the active site of Als3, while the nitro group interact with Tyr21 via hydrogen bonds [120].

Three *N’*-(1-(3-hydroxynaphthalen-2-yl)ethylidene)sulfonohydrazides (**33a**–**c**) were evaluated for antifungal and antibiofilm action against collection strains and clinical isolates. They surpassed fluconazole (MIC 128 μg/mL) in the case of clinical isolates of *C. albicans* and *C. krusei* (MIC 32 μg/mL). For the same strains, they inhibited biofilm formation at 32–64 μg/mL. All compounds downregulate hyphae-specific genes *hwp1*, *als3* and *ece1*, and **33b** and **33c** also reduced the expression of *sap5* genes, with propyl derivative being the most potent [121]. 

Schiff bases (**34a**–**e**) derivatives of 2-((hydrazinocarbonyl)methoxy)-4-phenyl-6-(2-thienyl)pyridine-3-carbonitril were synthesized and evaluated for antimicrobial and antibiofilm activity [122]. Compounds **34a** (2-((benzylidene-hydrazinocarbonyl)methyloxy)-4-phenyl-6-(2-thienyl)pyrid**i**ne-3-carbonitrile) and **34c** (2-((4-methoxy-benzylidene-hydrazinocarbonyl)methyloxy)-4-phenyl-6-(2-thienyl)pyridine-3-carbonitrile) were moderately active against *E. coli* planktonic and biofilm forms (IR 64.81, 64.61%, BI 78.75, 73.67%, respectively). The isatin derivative **34e** presented moderate antistaphylococcal activity and antibiofilm activity against *P. aeruginosa* (inhibition ratios over 60%). The chloro derivative **34d** had reduced or no effect [122].

A series of six (*EZ*)-*N’*-benzylidene-(2*RS*)-2-(6-chloro-9*H*-carbazol-2yl)-propanhydrazides (**35a**–**f**) were synthesized and tested for antibacterial, antifungal and antibiofilm activities [123]. Gram-positive bacteria (*S. aureus*, *E. faecalis*), as well as *C. albicans*, were sensitive to the action of the compounds, with MICs reaching 0.15–0.31 mg/mL for **3**5**a**, **35c** and **35d**. The antibiofilm activity was similar, with compound **35c** inhibiting *C. albicans* biofilm at 0.009 mg/mL while compound **35d** acted on *S. aureus*, *E. faecalis* (MBIC: 0.078 mg/mL). 4-chloro substitution was beneficial for antibacterial and antifungal activity and hydroxy enhanced antistaphylococcol action, whereas the 3,5-dichloro derivative **35f** was inactive [123].

**Figure 9:**
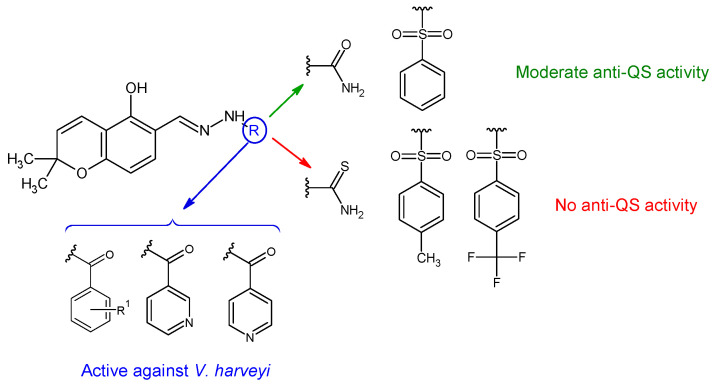
Structure–activity relationship for compounds **36**.

Hydrazones of 5-hydroxy-2,2-dimethyl-2*H*-chromene-6-carbaldehyde with different aryl, sulfonyl and non-aryl hydrazines were obtained and evaluated for QS inhibition and antibacterial activity [124]. Sulfonyl derivative ((*E*)-*N’*-((5-hydroxy-2,2-dimethyl-2*H*-chromen-6-yl)methylene)benzenesulfonohydrazide—**36f**) and semicarbazone (**36a**) exhibited moderate anti-QS activity (IC_50_ 22 μM, respectively 27 μM), but no antibacterial effect against *V. harveyi*. Substitution on the sulfonyl ring with hydrophobic groups (methyl, trifluoromethyl) or changing the urea to thiourea abolished anti-QS effect (Figure 9). Compounds **36d** (4-OH), **36c** (H), **36e** (2,4-diOH), **36i** and **36j** (pyridyl) presented antibacterial activity against *V. harveyi* (MIC 3.9, 7.8, 10.0, 10.0, 15.6 μM). Compound **36e** was the only one active against *S. aureus* (MIC 64 μg/mL) without effect on *E. coli* [124].

Starting from 4-[4-formyl-3-(2-naphthyl)pyrazol-1-yl]benzoic acid [126] and 4-[3-(7-fluoro-2-oxo-3,8a-dihydrochromen-3-yl)-4-formyl-pyrazol-1-yl]benzoic acid [125], two series of hydrazones have been obtained. The selected derivatives (**37a**–**d**, **38a**–**e**) exhibited antimicrobial activity against Gram-positive bacteria comparable to vancomycin (MIC 0.195-3.125 μg/mL). **37b** and **37c** inhibited *A. baumannii* as well. All compounds had the ability to inhibit *S. aureus* biofilm. **37a**,**c**,**d** reached over 85**%** inhibition and **38c** over 90% inhibition at 1/2 MIC, which is better than vancomycin (>60% inhibition at 1/2 MIC). They also disrupted the preformed biofilm—**37a**–**c** over 90% and **38b**,**e** over 70% at 1/2MIC.

Schiff base derivatives of androstane-1,4-diene-3,17-dione, thiosemicarbazone (**39a**) and isonicotinoylhydrazone (**39b**) presented antifungal and fungal biofilm inhibition properties. Both compounds surpassed ketoconazole (MIC 0.20–1.00 mg/mL) in some instances in terms of antifungal action, with thiosemicarbazone **39a** being the most potent. Compound **39b** also presented a higher binding affinity towards CYP51 of *C. albicans* than ketoconazole, interacting with Fe of heme. However, they were inferior biofilm inhibitors (ketoconazole: BI 25–55%), with compound **39a** performing slightly better than **39b** [127]. 

5-Nitro-2-thiophenecarbaldehyde *N*-[(*E*)-(5-nitrothienyl)methylidene)hydrazone (**40**) was evaluated for antistaphylococcal activity [128]. It inhibited Pan-S *S. aureus* at 0.5–2.0 μg/mL, VRSA and MRSA. Exposing the biofilm to this compound for 24 h led to a noteworthy (*p* < 0.05) decrease in the integrity of *S. aureus* biofilm at a concentration 4× MIC. The findings indicate that this hydrazone can impact *S. aureus* biofilm integrity even at concentrations 10–40× MIC. Additional research is necessary to gain a deeper understanding of the mechanism behind the disruption of *S. aureus* biofilm and potential interactions with biofilm-targeting properties of **40** and other antimicrobials available in clinical settings. 

(*E*)-1*H*-indole-3-carbaldehyde *O*-(4-chlorobenzyl)oxime, (*E*)-1*H*-indole-3-carbaldehyde *O*-(4-bromobenzyl) oxime (**41a**,**b**) and (*E*)-1-(1*H*-indol-3-yl)ethan-1-one O-(3,4-dichlorobenzyl)oxime (**41c**) presented antistaphylococcal activity (1–8 μg/mL) against standard and drug resistant strains (VRSA, MRSA). Biofilm inhibition capacity was reduced (10% at 1–10× MIC) comparable to references (levofloxacin and vancomycin) [129]. 

**Figure 10:**
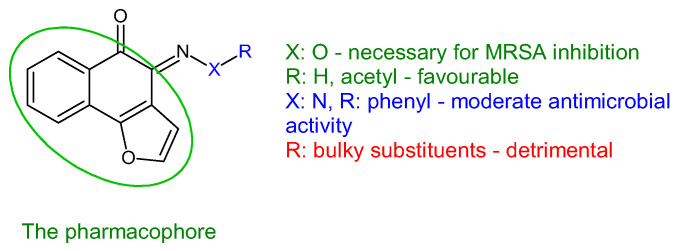
Structure–activity relationship for compounds **43**.

Antistaphylococcal furanoquinone derivatives (oximes, hydrazones) (**42**, **43**) were synthesized starting from naphto[2,3-b]furan-4,9-dione and naphto[1,2-b]furan-4,5-dione [130]. Based on biological evaluation, structure–activity relationships revealed that naphto[1,2-b]furan-4,5-dione is essential for activity, while linear furanoquinones are inactive. Oxime group (X: O) is necessary for MRSA inhibition, with small radicals (R: H- **43a**, COCH_3_- **43b**) being favoured over bulky substituents. Phenyl radicals coupled with hydrazine linker (X: N) showed moderate activity and were inferior to oximes (Figure 10). Thus, (*Z*)-4-(hydroxyimino)naphtho[1,2-b]furan-5(4*H*)-one (**43a**) and (*Z*)-4-(acetoxyimino)naphtho[1,2-b]furan-5(4*H*)-one (**43b**) were the most active of the series, being active against planktonic and sessile forms of MRSA. They exhibited bactericidal action against *S. aureus* standard strains, drug resistant strains and clinical isolates. They were able to penetrate MRSA biofilm and completely inhibit bacteria outside the matrix at 100 μg/mL, surpassing cetylpyridinium chloride. Bacteria inside the matrix were less susceptible, with a reduction of 4-log CFU being observed for hydroxy at 100 μg/mL. Biofilm height was reduced to half by both compounds. They also presented activity against MRSA-infected wounds with minimum skin irritation. The mechanism of action seemed to be inhibition of DNA gyrase (**43a**, **43b**) and RNA polymerase (**43a**) [130].

2-Methyl-l1-hydroxyimino-6,11-dihydrodibenzo[b,e]thiepin-5,5-dioxide (**44a**) and 2-methyl-l1-hydroxyimino-6,11-dihydrodibenzo[b,e]thiepin-5,5-dioxide (**44b**) [131] demonstrated microbicidal activity against the Gram-negative, non-fermentative *A. baumanii*. These oximes effectively hindered the adherence ability of *C. albicans* strains to inert substrata at a concentration of 250 µg/mL. Additionally, they displayed notable antibiofilm activity against the Gram-negative, non-fermentative bacilli *P. aeruginosa* and *A. baumanii*. Molecular modelling suggests that these compounds may interfere with the synthesis of quorum sensing molecules, specifically N-acyl-l-homoserine lactones, utilized by Gram-negative strains as their potential targets. It’s worth noting that despite the absence of fungicidal activity, compounds **44a** and **44b** exhibited inhibitory effects on the development of fungal biofilms.

Tetrahydroberberine, a natural alkaloid, was combined with metronidazole, a narrow-spectrum antimicrobial, and with oxime fragments to yield a series of derivatives (**45a**–**j**). These derivatives were subsequently tested for their antimicrobial and antibiofilm activities [55]. The hybrids demonstrated enhanced potency and a broader spectrum in comparison to berberine and metronidazole. The antimicrobial activity was influenced by the radical R of the oxime component. Linear alkyl groups (methyl to hexyl, **45b**–**h**) had a detrimental effect, whereas branched tert-butyl (**45f**), unsaturated allyl (**45i**) and benzyl (**45j**) were found to be beneficial. Simple oxime **45a** (9-(2-hydroxy-3-(2-methyl-5-nitro-1*H*-imidazol-1-yl)propoxy)-10-methoxy-5,8,13,13a-tetrahydro-6*H*-[1,3]dioxolo[4,5-g]isoquinolino[3,2-a]isoquinoline-12-carbaldehyde oxime) was active against Gram-positive bacteria (*S. aureus*, *E. faecalis*), Gram-negative bacteria (*E. coli*, *P. aeruginosa*, *A. baumannii*) and fungi *C. albicans*, *C. parapsilosis*, *A. fumigatus* (MIC 0.029–0.058 mM). Compound **45j** (9-(2-hydroxy-3-(2-methyl-5-nitro-1*H*-imidazol-1-yl)propoxy)-10-methoxy-5,8,13,13a-tetrahydro- 6*H*-[1,3]dioxolo[4,5-g]isoquinolino[3,2-a]isoquinoline-12-carbaldehyde O-benzyl oxime) had remarkably low MIC values (0.024–0.199 mM), especially on Gram-negative strains, *P. aeruginosa* in particular (0.024 mM), surpassing in some instances norfloxacin. It was also able to reduce *P. aeruginosa* biofilm in a dose-dependent manner (45% inhibition at 8× MICs) and seemed to act against bacterial cell membrane. Regarding antifungal assay, **45j** inhibited all fungal strains except *C. parapsilosis* (MIC 0.024–0.199 mM) [132].

## 4. Materials and Methods

The literature survey was conducted across four databases (Web of Science, ScienceDirect, Scopus and Reaxys). The primary keywords employed were (“imine” OR “azomethine” OR “Schiff base” OR ”hydrazone” OR ”oxime”) AND (“biofilm” OR “biofilm inhibitor” OR “antibiofilm” OR “anti-biofilm”), covering a ten-year span (2013–2023) with search parameters adjusted for each database. Inclusion criteria comprised of the English language, original research articles, antibiofilm evaluation and a focus on small molecules. The emphasis was on Schiff bases acting as antimicrobials and antibiofilm agents with medical applications, leading to the exclusion of metal complexes, Schiff base polymers, antibiofouling agents and other categories. It is noteworthy that while the scientific literature extensively covers antibacterial evaluations of Schiff bases, only a limited number of studies tested their antibiofilm potential. Consequently, numerous articles had to be excluded. Following duplication removal, title and abstract screening, full-text screening, eligibility analysis and cross-checking, the most relevant articles were selected and reviewed.

## 5. Conclusions

Although Schiff bases have demonstrated their antimicrobial efficacy, their potential as small molecules to inhibit biofilm formation remains an area that requires further exploration. A significant challenge is that while the antimicrobial screening is a routine practice for assessing the biological potential of Schiff bases, antibiofilm assays are not consistently taken into consideration.

Imine moiety may be included in a molecule for several reasons. For example, it may serve as a link between two structures resulting in hybrid compounds. It may also be used in isostere substitution of carbonyl group—amide or ether. Imine groups are polar and may form hydrogen bonds with aminoacids from the active sites as they are important for biological activity.

There are several cases where the antimicrobial activity of Schiff bases and their derivatives were superior compared to the parent compound, even turning bacteriostatic action into bactericidal. The spectrum may be reduced to Gram-positive like *Staphylococcus aureus* for halogenated salicylaldehyde Schiff bases or *Enterococcus faecalis* for benzensulfonyl thiazoloimines. Some Schiff bases have a broad spectrum, including Gram-negatives such as *P. aeruginosa*, *K. pneumoniae*, *A. baumannii* and fungi.

The antibiofilm potential is variable and usually moderate compared to antimicrobial activity of the same compounds or references. In general, the most active antimicrobials were evaluated for biofilm inhibition. There are also Schiff bases, as we have shown in this article, which presented a remarkably biofilm inhibition.

According to the findings outlined in this article, it can be confirmed that Schiff bases serve as a molecular framework worthy of investigation for their potential antibiofilm properties. The prospective areas for future research include synthesizing hybrid compounds within this class, utilizing known antimicrobial agents as starting materials and testing the antibiofilm efficacy of certain Schiff bases recognized for different therapeutic applications, which has aims to reposition these substances for antibiofilm purposes. Furthermore, Schiff bases with antimicrobial and antibiofilm properties, originating from natural products, present a promising avenue for identifying potential lead molecules. Finally, it is crucial to conduct further studies on the Schiff bases that have demonstrated antibiofilm efficacy. These investigations should focus on unraveling their mechanisms of action, exploring potential synergistic relationships, assessing therapeutic potency and ensuring safety.

Thus, Schiff bases still remain an open door for the antibiofilm research.

## Figures and Tables

**Table 1:** Classical Schiff bases, oximes and hydrazones with antibiofilm-antimicrobial activity.

Compounds	Biological Assay/Microorganism	Observations	Ref.
**Classical Schiff Bases**			
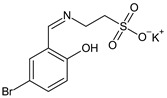 Taurine-5-Bromosalicylaldehyde Schiff base	**Antibacterial screening**: *Staphylococcus aureus* ATCC 43300, *Mycobacterium smegmatis* mc^2^155	SA: MIC 32 μg/mL MS: MIC > 60 μg/mL	[97,98]
	**Antibiofilm screening**: *S. aureus* ATCC 43300, *M. smegmatis* mc^2^155	Biofilm inhibition SA: MBIC 8 μg/mL	
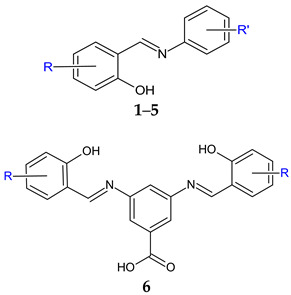 R’: 4-COOH (**1**); 3-COOH (**2**); 4-COOCH_3_ (**3**); 4-COOCH_2_CH_3_ (**4**); 4-CONHC_6_H_5_ (**5**) R: 3-I-5-Cl (**a**); 3,5-diI (**b**)	**Antibacterial screening**: *S. aureus* ATCC 29213, methicillin-resistant *S. aureus* ATCC 43300, *S. epidermidis*, clinical isolate 143-2016, *Enterococcus faecalis* ATCC 29212, *Escherichia coli* ATCC 25922, *Klebsiella pneumoniae* ATCC 10031, *Serratia marcescens*, clinical isolate 62-2016, *Pseudomonas aeruginosa* ATCC 27853.	Gram-positive bacteria—susceptible MIC ≥ 7.81 μM (SA, MRSA) MIC ≥ 15.62 μM (SE, EF)	[99]
	**Antimycobacterial screening**: *Mycobacterium tuberculosis* 331/88 (H_37_Rv), *M. avium 330/88*, *M. kansasii* 6509/96	No activity	
	**Antifungal screening**: *Candida albicans* ATCC 24433, *Candida krusei* ATCC 6258, *Candida parapsilosis* ATCC 22019, *Candida tropicalis* ATCC 750, *Aspergillus fumigatus* ATCC 204305, *Aspergillus flavus* CCM 8363; *Lichtheimia corymbifera* CCM 8077, *Trichophyton interdigitale* ATCC 9533	CA, TI—susceptible MIC ≥ 3.90 μM (TI) MIC ≥ 7.81 μM (CA)	
	**Antibiofilm screening**: methicillin-resistant *S. aureus* ATCC 43300, *S. epidermidis* ATCC 1228	**3b**—MBIC 781.25-1562.5 μg/mL, MBEC 1562.5–3125.0 μg/mL (MRSA) MBIC 781.25–1562.5 μg/mL, MBEC > 1562.5 μg/mL (SE)	
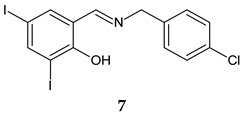 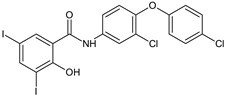 Rafoxanide	**Antibacterial screening**: methicillin-resistant *S. aureus* ATCC 43300, and clinical isolates 131/16, 138/16, 153/16; methicillin-sensitive *S. aureus* 136/16, 141/16, 154/16*;* vancomycin-resistant *S. aureus* 203/19 NIPH, CCM 1767; *S. epidermidis* ATCC 1228; vancomycin-resistant *E. faecium* 198/16	Bactericidal MIC 15.625–62.5 μM (SA) MIC 62.5–125 μM (EF)	[100]
	**Antibiofilm screening**: methicillin-resistant *S. aureus* ATCC 43300; *S. epidermidis* ATCC 12228	MBIC 62.216–124.432 μg/mL, MBEC 124.432–248.863 μg/mL (MRSA), MBIC 31.108–62.216 μg/mL, MBEC 124.432–248.863 μg/mL (SE)	
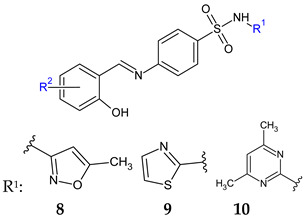 R^2^: 3,5-diCl (**a**), 3-Br-5-Cl (**b**), 3-I-5-Cl (**c**), 3,5-diI (**d**), 6-Cl (**e**), H (**f**)	**Antibacterial screening**: *S. aureus* ATCC 2913, CCM 4223; methicillin-resistant *S. aureus* ATCC 43300, CCM 4750; *S. epidermidis* H 6966/08; *S. epidermidis* H2232, *S. epidermidis* D7944 (clinical isolate), *S. epidermidis* H2232 (clinical isolate); *S. hominis H2202* (clinical isolate)*; E. faecalis* ATCC 29212, CCM 4224; *E. coli* ATCC 25922, CCM 3954; *K. pneumoniae* D 11750/08; ESBL-positive *K. pneumoniae* J 14368/08; *P. aeruginosa* ATCC 27853, CCM 3955.	Gram-positive bacteria—susceptible Bactericidal **8e**, **8f**, **9d**: MIC 31.25 μM (EF) **9d**, **10d**: MIC 15.62 μM (SA, MRSA, SE), **10d**: MIC 3.91 μM (SE H2202)	[101]
	**Antibiofilm screening**: methicillin-resistant *S. aureus* ATCC 43300, *S. epidermidis* ATCC 1228	No biofilm disruption **10a:** MBIC 390.6–781.25 μM, MBEC > 3462 μM (MRSA, SE)	
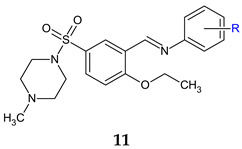 R: H (**a**), 2-CF_3_ (**b**), 3-CF_3_ (**c**), 2-OH (**d**), 4-OH (**e**), 4-OCH_3_ (**f**)	**Antibacterial screening**: *B. subtilis* NCIM-2063, *S. aureus* NCIM-2901, *E. coli* NCIM-2256, *P. aeruginosa* NCIM-2036	**11b**—MIC 35.7 μg/mL (SA) **11c**—MIC 84.0 μg/mL (EC) **11f**, **11a**—MIC 39.0, 40.0 μg/mL (PA)	[102]
	**Antifungal screening**: *C. albicans* NCIM-3471	**11d**, **11c**, **11e**—MIC 39.6, 45.0, 47.2 μg/mL	
	**Antibiofilm screening: ** *C. albicans* NCIM-3471	**11d**, **11a**, **11c**, **11e**—IC_50_ 31.4, 32.1, 37.2, 39.5 μM	
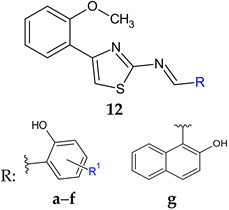 R^1^: H (**a**), 3-CH_3_ (**b**), 4-CH_3_ (**c**), 5-CH_3_ (**d**), 3-OCH_3_ (**e**), 5-Br (**f**)	**Antibacterial screening**: *B. subtilis* NCIM 2063; *E. coli* NCIM 2931	**12f**, **12g**—MIC 25μg/mL, MBC 50 μg/mL (BS) **12g**—MIC = MBC 100 μg/mL (EC)	[103]
	**Antibiofilm screening**: *P. aeruginosa*	QS mediated mechanism	
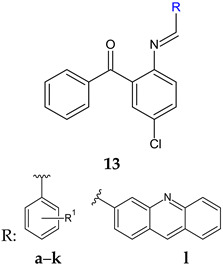 R^1^: 2-OH-4-OCH_3_ (**a**), 2-NO_2_-5-OH (**b**), 3-Br-4-F (**c**), 2-OH (**d**), 2-OH-5-F (**e**), 2-OH-5-Br (**f**), 2-OH-3-Br-5-Cl (**g**), 3-OH-4-OH (**h**), 3-Br-4-OH (**i**), 3-Cl-4-OH (**j**), 3-Br-4-OCH_3_ (**k**)	**Antibacterial screening**: *S. mutans* ATCC 25175, *S. aureus* ATCC 43300, *Proteus mirabilis* ATCC 12453, *K. pneumoniae* ATCC 13882	**13g**—MIC 20 μg/mL (SM), 36.22 μg/mL (SA), 144.9 μg/mL (PM) **13l**—MIC > 58.1 μg/mL (PA) **13f**—MI**C** 79.45 μg/mL (KP)	[104]
	**Antibiofilm screening**: *S. mutans* ATCC 25175, *S. aureus* ATCC 43300, *P. mirabilis* ATCC 12453, *K. pneumoniae* ATCC 13882	MBIC < 100 μg/mL **13i**, **13k**, **13g**: disruption of SA biofilm **13i**: Disruption of preformed biofilm (PM)	
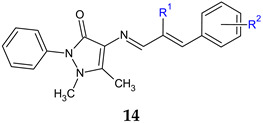 R^1^: Br, R^2^: H (**a**), R^1^: H, R^2^: 4-NO_2_ (**b**), R^1^: H, R^2^: 3-OCH_3_-4-OCOCH_3_ (**c**)	**Antibacterial screening**: *S. aureus* ATCC 25923, *E. faecalis* ATCC 29212, *Salmonella enterica* ATCC 14028, *Klebsiella ozaenae* (clinical isolate), *Enterobacter gergoviae* (clinical isolate) *P. aeruginosa* ATCC 27853	**14a**: MIC 15.60 μM (*E. gergoviae*), 31.25 μM (*S. enterica*), 62.5 μM (*K. ozonae*, SA), 125 μM (EF) **14b**: 250 μM (EF, CT) **c**: 250 μM (EF)	[105]
	**Antifungal screening:** *C. albicans* (clinical isolate), *C. krusei* (clinical isolate), *C. tropicalis* (clinical isolate), *C. glabrata* (clinical isolate)	**14a**: MIC 15.60–62.50 μM **14b**: 250 μM	
	**Antibiofilm screening**: *S. aureus* ATCC 25923, *E. faecalis* ATCC 29212, *C. tropicalis* (clinical isolate)	**14a**: BI 82.77% (SA), 75.69% (EF), 90.41% (CT) **14b**: BI 76.63% (EF)	
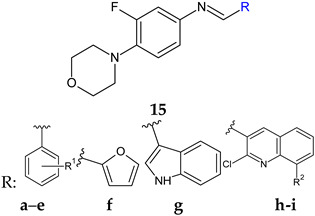 R^1^: H (**a**), 4-F (**b**), 2,6-diCl (**c**), 3,4-diOH (**d**), 4-diOCH_3_ (**e**) R^2^: H (**h**), CH_3_ (**i**) 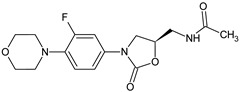 Linezolid	**Antibacterial screening**: *B. subtilis* NCIM-2063, *E. coli* NCIM-2256, *P. aeruginosa* NCIM-2036	**15h**—MIC 2.5 ± 0.15 **15i**—MIC 3.5 ± 0.18 μg/mL (PA)	[106]
	**Antibiofilm screening**: *P. aeruginosa* O1	PqsD inhibition **15h**—IC_50_ 12.97 ± 0.33 μM **15i**—IC_50_ 15.63 ± 0.20 μM	
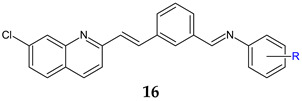 R: H (**a**), 4-Cl (**b**), 2-CH_3_ (**c**), 4-CH_3_ (**d**), 2-CF_3_ (**e**), 3-CF_3_ (**f**), 4-CF_3_ (**g**), 4-OCH_3_ (**h**), 3-NO_2_ (**i**), 4-NO_2_ (**j**)	**Antibacterial screening**: *B. subtilis* NCIM-2063, *S. aureus* NCIM-2901, *E. coli* NCIM-2256, *P. aeruginosa* NCIM-2036	**16b**: MIC 45 μg/mL (EC) **16g**: MIC 91.5 μg/mL **(**PA) **16e**: MIC 55.3 μg/mL (SA)	[107]
	**Antifungal screening**: *C. albicans* NCIM-3471	**16b**: MIC 94.2 μg/mL **16a**: MIC 98.8 μg/mL	
	**Antibiofilm screening**: *C. albicans* NCIM-3471	Als-3 adhesin inhibition **16g**: IC_50_ 51.2 μM **16b**: IC_50_ 66.2 μM	
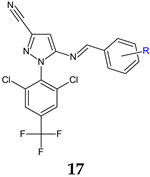 R: 4-NO_2_ (**a**), 4-COOH (**b**), 3,4-diOH (**c**), 2,4-diOCH_3_ (**d**), 2,4-diCl (**e**), 3,4-diOCH_3_ (**f**), 2,5-diOCH_3_ (**g**), 3-CN (**h**), 4-Br (i), 4-Cl (**j**)	**Antifungal screening**: *C. albicans*	**17i**: MIC 42.6 μg/mL	[108]
	**Antifungal screening**: *C. albicans*	**17i**: IC_50_ 41.5 μM	
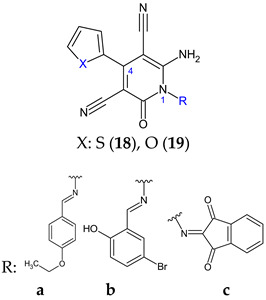	**Antibacterial screening**: methicillin sensitive *S. aureus* ATCC 25923, methicillin resistant *S. aureus* ATCC 43300, *E. coli* ATCC-25922, *K. pneumoniae* ATCC-700603, *P. aeruginosa* ATCC-2785, *Acinetobacter baumannii* ATCC-19606	**19b**: MIC 62.5 μg/mL (MRSA) **19a**: MIC 125 μg/mL (EC), 15.6 μg/mL (KP) **18c**: MIC 62.5 μg/mL (PA) **18a**, 19a, 18e: MIC 3.9 μg/mL (AB)	[109]
	**Antifungal screening**: *C. albicans* ATCC-10231	**18c**: MIC 15.6 μg/mL	
	**Antibiofilm screening**: methicillin resistant *S. aureus* ATCC 43300, *E. coli* ATCC-25922, *P. aeruginosa* ATCC-2785, *C. albicans* ATCC-10231	Downregulation of *Las*R **19b**: BI 64.7 ± 1.85% (MRSA) **18b**, **19c**: BI 63.8% (EC) **19c**: BI 45.4 ± 1.30% (PA), 75.0 ± 0.51% (CA)	
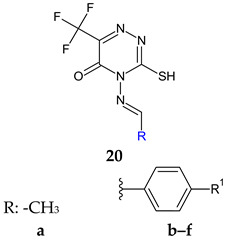 R^1^: F (**b**), Cl (**c**), Br (**d**), NO_2_ (**e**), CF_3_ (**f**)	**Antibacterial screening**: *B. subtilis* ATCC 6633, *S. aureus* NRRL B-767, *Salmonella typhi*, *E. coli* ATCC 25955	**20b**: MIC 3.90 μg/mL (SA, EC) **20a**: MIC 7.81 μg/mL (ST)	[110]
	**Antifungal screening**: *A. niger* *A. flavus*	**20c**, **20f**: MIC 3.90 μg/mL (AF) **20f**: MIC 15.62 μg/mL (AN)	
	**Antibiofilm screening**: *S. aureus*, *E. coli*	**20b**: BI 72.34% (SA), 87.38% (EC)	
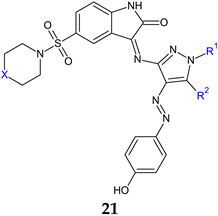 **a**: X: CH_2_, R^1^: H, R^2^: OH; **b**: X: CH_2_, R^1^: phenyl, R^2^: NH_2_; **c**: X: N(CH_3_), R^1^: phenyl, R^2^: NH_2_; **d**: X: CH_2_, R^1^: benzoyl, R^2^: NH_2_; **e**: X: N(CH_3_), R^1^: benzoyl, R^2^: NH_2_; **f**: X: N(CH_3_), R^1^: benzoyl, R^2^: OH	**Antibacterial screening:** *S. aureus* ATCC 6538, *E. faecalis* ATCC 29212, *E. coli* ATCC 35218, *P. aeruginosa* ATCC 27853	Bactericidal **21b:** 56.07 μM (SA, EF, PA), 112.16 μM (EC) **21d**: 53.45 μM (EC, EF), 106.91 (SA, PA)	[111]
	**Antifungal screening:** *C. albicans* ATCC 90028	Fungicidal **21d**: 106.91 μM	
	**Antibiofilm screening: ** *S. aureus* ACL51 (MRSA)	**21d**: BI 89.9 ± 4.7, 89.7 ± 9, 70.8 ± 2.3% at 0.03, 0.015, 0.007 mg/mL	
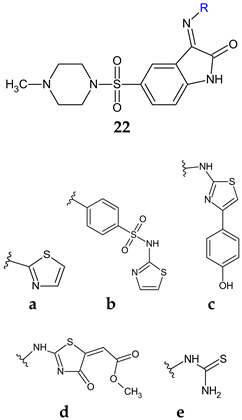	**Antibacterial screening:** *S. aureus* ATCC 25923, *B. subtilis* ATCC 6051, *E. faecalis* ATCC 29212, *E. coli* ATCC 35218, *P. aeruginosa* ATCC 27853, *S. typhimurium*ATCC14028	**22c**: MIC 1.9 (EC), 7.8 (ST), 15.6 (SA, PA), 31.2 μg/mL (BS)	[112]
	**Antifungal screening:** *C. albicans* ATCC10213	**22d**: MIC 31.2 μg/mL	
	**Antibiofilm screening: ** *S. aureus* ATCC 29213, *P. aeruginosa* ATCC 9027	**22b**: BI_50_ 1.95 μg/mL (SA) **22a**, **22c**, **22d**: BI_50_ 15.6 μg/mL (SA) **22c**: BI_50_ 7.8 μg/mL (PA)	
	**Anti-quorum sensing**: *E. faecalis* ATCC 29212	**22c**: 83.9, 73.0 and 64.9% fsr system inhibition at 3.9, 1.9 and 0.9 µg/mL	
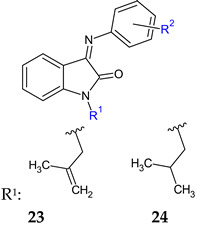 R^2^: 4-COOH (**a**), 2-COOH-4-Cl (**b**)	**Antibacterial screening**: *B. subtilis* ATCC10400, *S. aureus* ATCC29213	**23a**: MIC 0.09 mmol/L (SA, BS) **23b**: MIC 0.181 mmol/L (SA, BS)	[113]
	**Antibiofilm screening**: *S. aureus* ATCC29213, methicillin-resistant *S. aureus* ATCC35501	**23a**, **23b**: BI 55%	
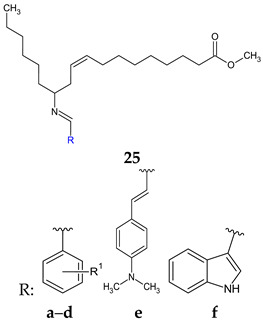 **R^1^:** 4-Cl (**a**), 4-N(CH_3_) (**b**), 4-OH-3-OCH_3_ (**c**), 4-OH-3,5-(OCH_3_)_2_ (**d**)	**Antibacterial screening**: *Micrococcus luteus* MTCC 2470, *S. aureus* MTCC 96, *S. aureus* MLS-16 MTCC 2940, *B. subtilis* MTCC 121, *E. coli* MTCC 739, *P. aeruginosa* MTCC 2453, *Klebsiella planticola* MTCC 2453	Gram-positive bacteria—susceptible (SA, BS) **25a**: MIC 9.0 μM, MBC 9–18 μM **25c**: MIC 17.4 μM, MBC 35 μM, **25d**: MIC 16.4 μM, MBC 16.4–32.8 μM	[114]
	**Antibiofilm screening**: *S. aureus* MTCC 96, *S. aureus* MLS-16 MTCC 2940, *B. subtilis* MTCC 121	**25a**: IC_50_ 4.3–6 μM **25d**: IC_50_ 6.5–8.6 μM **25c**: IC_50_ 8.0–9.4 μM (SA) **25f**: IC_50_ 7.3–9.5 μM (SA)	
**Oximes and Hydrazones**			
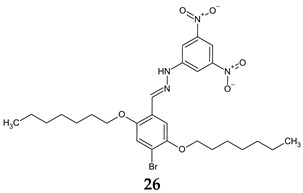	**Antibacterial screening**: *S. aureus* ATCC 25923, *E. faecalis* ATCC 29212, *E. coli* ATCC 25922, *P. aeruginosa* ATCC 27853	MIC 0.625 mg/mL (EF) MIC 1.25 mg/mL (SA) MIC 2.50 mg/mL (PA, EC)	[115]
	**Antifungal screening:** *C. albicans* ATCC 10239, *C. tropicalis* ATCC 13803	MIC 0.625 mg/mL	
	**Antibiofilm screening:** *S. aureus* ATCC 25923, *E. faecalis* ATCC 29212, *E. coli* ATCC 25922, *P. aeruginosa* ATCC 27853, *C. albicans* ATCC 10239, *C. tropicalis* ATCC 13803	SA: BI 24.30–72.24% (MIC/4–MIC) EF: BI 23.41–49.55% PA: 12.50–28.30% CA: 10.26–25.83% CT: 23.90–40.15% (MIC/2–MIC)	
	**Violacein inhibition:** *C. violaceum* CV12472	5.7–100% (MIC/32–MIC)	
	**QS inhibition:** *C. violaceum* CV026	7.0–10.5 mm (MIC/2–MIC)	
	**Swarming motility inhibition:** *P. aeruginosa* PA01	14.4–45.7% (MIC/4–MIC)	
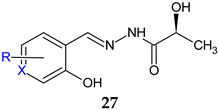 X = CH; R: H (**a**), 3-OCH_3_ (**b**), 5-Br (**c**), 5-I (**d**), 5-NO_2_ (**e**), 5-OH (**f**) X = N; R: 3-CH_3_-6-CH_2_OH (**g**)	**Antibacterial screening**: *S. aureus* PTCC 1112, *S. pneumonia* PTCC 1240, *E. coli* ATCC 25922, *P. aeruginosa* PAO1	**27e**, **27g**: MIC 64 μg/mL (SA, EC)	[116]
	**Antibiofilm screening**: *P. aeruginosa* PAO1	**27e**, **27g**: Significantly reduction (1/16 and 1/4 MIC)	
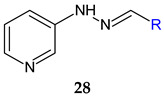 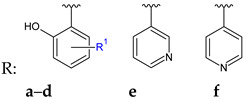 R^1^: 3-OH (**a**), 3-OCH_3_ (**b**), 4-OCH_3_ (**c**), 5-NO_2_ (**d**)	**Antibacterial screening**: *A. baumannii* ATCC 19606 *A. baumannii* clinical isolates 1–4	MIC 25-200 μg/mL **28d**: 25 μg/mL	[117]
	**Antibiofilm screening**: *A. baumannii* ATCC 19606 *A. baumannii* clinical isolates 4	**28a**–**f**: Biofilm inhibition (MIC-2× MIC) **28a**, **28d**: Biofilm disruption (12.5 μg/mL) **28a**: BEC_50_ 28.2 μg/mL **28d**: BEC_50_ 12.8 μg/mL	
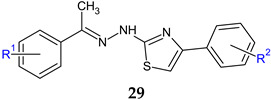 R^1^: H, R^2^: H (**a**); R^1^: H, R^2^: 4-OCH_3_ (**b**); R^1^: H, R^2^: 4-CH_3_ (**c**); R^1^: 2,4-diF, R^2^: 4-NO_2_ (**d**); R^1^: 2,4-diF, R^2^: 4-CN (**e**)	**Antibiofilm screening**: *C. albicans*, clinical isolate	Upregulation of *bcy1*, *nrg1*, *tup1* Downregulation of *als3*, *hwp1*, *ras1* **29a**–**c**: 100 μg/mL **29d**–**e**: 50 μg/mL	[118]
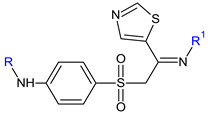 R: H (**30**), -CO-CH_3_ (**31**) R^1^: OH (**a**), *n*-OC_6_H_13_ (**b**), 4-chlorobenzyloxy (**c**), 2,4-dinitrophenylamino (**d**), 4-carboxyphenylamino (**e**), pyridine-2-carboxamido (**f**), pyridine-3-carboxamido (**g**), yridine-4-carboxamido (**h**)	**Antibacterial screening**: *S. aureus* ATCC 25923, *S. aureus* ATCC 29213, methicillin-resistant *S. aureus* N315, *E. faecalis* p1-2007226001, *E. faecalis* p1-2007225053, *K. pneumon*iae; *E. coli* ATCC 25922, *P. aeruginosa* ATCC 27853, *A. baumannii*	Gram-positive bacteria—susceptible **31f**: MIC 1–4 μg/mL (EF)	[119]
	**Antibiofilm screening**: *E. faecalis*	**31f**: BI 35% (6× MIC)	
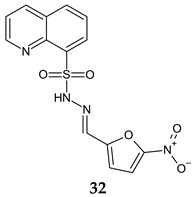	**Antibacterial screening:** *S. aureus* ATCC 25923, *S. aureus* ATCC 43300, *E. coli* NCTC 9001, *Listeria monocytogenes* NCTC 11994	MIC 125 μg/mL (SA)	[120]
	**Antifungal screening**: *C. albicans* ATCC 10231, *C. albicans* ATCC 24433, *C. parapsilosis* ATC 22019, *C. krusei* ATCC 6258, *C. glabrata* ATCC 2001, clinical isolates (veterinary samples)	MIC 31.2 μg/mL (CA ATCC 10231, CP)	
	**Anti-filamentation assay:** *C. albicans* ATCC 10231	24.96 μg/mL— inhibition	
	**Antibiofilm screening**: *C. albicans* ATCC 10231	31.2 μg/mL—38% inhibition	
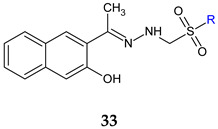 R: C_2_H_5_ (**a**), n-C_3_H_7_ (**b**), n-C_4_H_9_ (**c**)	**Antifungal and antibiofilm screening:** *C. glabrata* ATCC 90030 *C. krusei* ATCC 6258 *C. krusei* clinical isolates *C. albicans* ATCC 10231 *C. albicans* clinical isolates *C. parapsilosis* ATCC 22019 *C. parapsilosis* clinical isolates *C. tropicalis* NRRLY-12968 *C. lusitaniae* clinical isolates	MIC 32–64 μg/mL BIC 32–64 μg/mL (CA, CT, CK) BIC 64–128 μg/mL (CP, CL) Downregulation of *hwp1*, *als3*, *ece1* and *sap5* genes	[121]
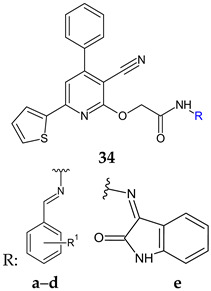 R^1^: H (**a**), 4-CH_3_ (**b**), 4-OCH_3_ (**c**), 4-Cl (**d**)	**Antibacterial screening**: *S. aureus*, *B. subtilis*, *E. coli*, *P. aeruginosa*	**34a**: IR 64.81% (EC) **34c**: IR 64.61% (EC)	[122]
	**Antibiofilm screening**: *S. aureus*, *B. subtilis*, *E. coli*, *P. aeruginosa*	**34a**: BI 78.75% (EC)	
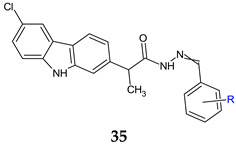 R = 2-OH (**a**), 2-OH-3-OCH_3_ (**b**), 2-OH-5-OCH_3_ (**c**), 4-Cl (**d**), 2,6-diCl (**e**), 3,5-diCl (**f**)	**Antibacterial screening**: *S. aureus* ATCC 25923, *E. faecalis* ATCC 29212, *E. coli* ATCC 25922, *P. aeruginosa* ATCC 27853	**35d**: MIC 0.15 mg/mL (EF)	[123]
	**Antifungal screening**: *C. albicans* ATCC 10231	**35a**, **35c**, **35d**: MIC 0.31 mg/mL	
	**Antibiofilm screening**: *S. aureus* ATCC 25923, *E. faecalis* ATCC 29212, *E. coli* ATCC 25922, *P. aeruginosa* ATCC 27853, *C. albicans* ATCC 10231	**35d**: MBIC 0.078 mg/mL (EC, SA) **35c**: MBIC 0.009 mg/mL (CA)	
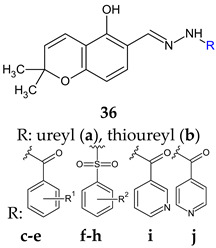 R^1^: H (**c**), 4-OH (**d**), 2,4-di-OH (**e**) R^2^: H (**f**), 4-CH_3_ (**g**), 4-CF_3_ (**h**)	**Antibacterial screening**: *Vibrio harveyi* BB120, *S. aureus* MW2, *E. coli*	**36d**: MIC 3.9 μg/mL (VH) **36e**: MIC 64 μg/mL (SA)	[124]
	**Anti-quorum sensing**: *V. harveyi* BB120	**36f**: IC_50_ 22 μM **36a**: IC_50_ 27 μM	
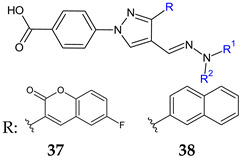 **37:** R^1^ = R^2^: C_6_H_5_ (**a**), R^1^: H, R^2^: 3-Cl-C_6_H_4_ (**b**), R^1^: H, R^2^: 3-Br-C_6_H_4_ (**c**), R^1^: H, R^2^: 4-CF_3_-C_6_H_4_ (**d**) **38:** R^1^ = R^2^: C_6_H_5_ (**a**), R^1^ = R^2^: CH_2_C_6_H_5_ (**b**), R^1^: H, R^2^: 2,5-F_2_-C_6_H_3_ (**c**), R^1^: H, R^2^: 2-F-3-Cl-C_6_H_3_ (**d**), R^1^: H, R^2^: 4-CF_3_-C_6_H_4_ (**e**)	**Antibacterial screening**: *S. aureus* ATCC 25923, *S. aureus* BAA-2312, *S. aureus* ATCC 33591, *S. aureus* ATCC 700699, *S. aureus* ATCC 33592, *S. epidermidis* 700296, *B. subtilis* ATCC 6623; *A. baumannii* ATCC 19606, *A. baumannii* ATCC BAA-1605, *A. baumannii* ATCC 747	**37a**–**d**: MIC 3.125–12.5 μg/mL (SA, SE, BS) **37b,c**: 6.25–25 μg/mL (AB) **38a**–**e**: 0.78–25 μg/mL (SA, SE, BS)	[125,126]
	**Antibiofilm screening**: *S. aureus* ATCC 25923	Biofilm inhibition **37a**,**c**,**d, 38a**–**c**: > 85% (1/2–2× MIC) Biofilm destruction **37a**–**c**: >90%, **38b**,**e**: > 70% (1/2–2× MIC)	
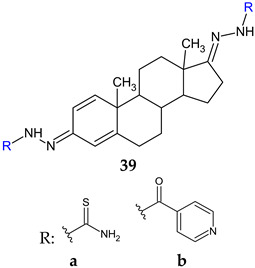	**Antifungal screening:** *A. fumigatus* ATCC 1022, *A. niger* ATCC 6275, *Trichoderma viride* IAM 5061, *Penicillium funiculosum* ATCC 36839, *Penicillium verrucosum* var. *cyclopium* (food isolates), *C. albicans* ATCC 10231	**39a**: 0.37 mg/mL **39b**: 0.37–0.75 mg/mL	[127]
	**Antibiofilm screening:** *C. albicans* ATCC 10231	**39a:** BI 33% (MIC), 18% (MIC/2-MIC/4) **39b**: BI 16% (MIC/2–MIC), 5% (MIC/4)	
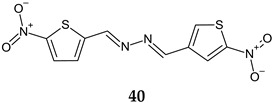	**Antibacterial screening**: *S. aureus* SA-1001, *S. aureus* ME-311, *S. aureus* VA13	MIC_50_ 0.5–4.0 μg/mL MIC_90_ 1–4.0 μg/mL Bactericidal	[128]
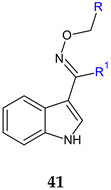 R^1^: H, R:4-Cl-C_6_H_4_ (**a**), R^1^: H, R:4-Br-C_6_H_4_ (**b**), R^1^: CH_3_, R:2,4-Cl_2_-C_6_H_4_ (**c**)	**Antibiofilm screening:** *S. aureus*	Altered biofilm integrity (10× MIC)	
	**Antibacterial screening**: *S. aureus* ATCC 29213, MRSA clinical isolates, VRSA clinical isolates	MIC 2–8 μg/mL **41c**: 1–4 μg/mL	[129]
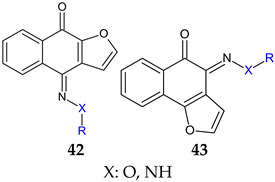 R: H, methyl, benzyl, acetyl, phenyl, 4-fluoro-phenyl, 4-methoxy-phenyl, 4-tolyl, methyl-sulfonyl 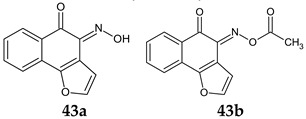	**Antibacterial screening**: Methicillin-resistant *S. aureus* ATCC 33591, *S. aureus* clinical isolates (KM-1, KM-5), vancomycin-intermediate *S. aureus* (KV-1, KV-5), *E. coli* ATCC 8739	**43a**: MIC 9.7–19.5 μg/mL, MBC 3.9–156 μg/mL **43b**: MIC 2.4–9.7 μg/mL, MBC 19.5–39 μg/mL	[130]
	**Antibiofilm screening**: Methicillin-resistant *S. aureus* ATCC 33591	Cell outside—complete inhibition at 100 μg/mL Cell inside— **43a**: 4-log CFU reduction at 100 μg/mL	
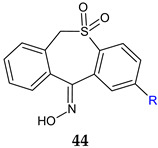 R: H (**a**), CH_3_ (**b**)	**Antibacterial screening** *B. subtilis* 12488 *A. baumannii* 221 *P. aeruginosa* 207	**44a:** 125 μg/mL (AB), 250 μg/mL (PA) **44b**: 250 μg/mL (BS, AB)	[131]
	**Antibiofilm screening**: *S. aureus* IC 13202 *B. subtilis* 12488 *A. baumannii* 221 *C. albicans* 101404 *C. albicans* IC249	**44a,b**: 125 μg/mL (AB) **44a**: 250 μg/mL (BS, SA, CA) **44b**: 250 μg/mL (CA)	
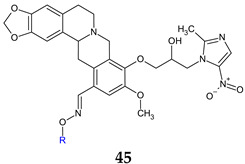 R: H (**a**), methyl (**b**), ethyl (**c**), propyl (**d**), butyl (**e**), t-butyl (**f**), pentyl (**g**), hexyl (**h**), allyl (**i**), benzyl (**j**)	**Antibacterial screening**: Methicillin-resistant *S. aureus*, *S. aureus* 25923, *S. aureus* 29213, *E. faecalis*, *K. pneumoniae*, *E. coli*, *E. coli* 25922, *P. aeruginosa*, *P. aeruginosa* 27853, *A. baumannii*	**45j**: MIC 0.024 (PA)-0.199 mM	[132]
	**Antifungal screening**: *C. albicans*, *C. albicans* ATCC 90023, *C. tropicalis*, *C. parapsilosis* 22019, *A. fumigatus*	**45j: ** MIC 0.024–0.199 mM (except *C. parapsilosis*)	
	**Antibiofilm screening**: *P. aeruginosa*	**45j:** 45% inhibition at 8× MIC	

## Data Availability

Not applicable.

## Abbreviations

AB	*Acinetobacter baumannii*
AF	*Aspergillus flavus*
AN	*Aspergillus niger*
ATCC	American Type Culture Collection
BC	*Bacillus subtilis*
BI	biofilm inhibitory percentage
BI_50_	concentration required for 50% inhibition of biofilm formation
BEC50	concentration required for 50% eradication of mature biofilm
CA	*Candida albicans*
CK	*Candida krusei*
CL	*Candida lusitaniae*
CP	*Candida parapsilosis*
CT	*Candida tropicalis*
CFU	colony-forming unit
DABA	3,5-diaminobenzoic acid
EC	*Escherichia coli*
EF	*Enterococcus faecalis*
EPS	extracellular polymeric substances
IAM	Japan Collection of Microorganisms
IC_50_	half maximal inhibitory concentration
IR	inhibition ratio
KP	*Klebsiella pneumoniae*
MABA	*m*-aminobenzoic acid
MBC	minimum bactericidal concentration
MBEC	minimum biofilm eradication concentration
MBIC	minimum biofilm inhibition concentration
MDR-TB	multidrug-resistant tuberculosis
MIC	minimum inhibitory concentration
MIC_50_	minimum concentration inhibiting growth of 50% tested strains
MIC_90_	minimum concentration inhibiting growth of 90% tested strains
MRSA	methicillin-resistant *Staphylococcus aureus*
MS	*Mycobacterium smegmatis*
NCTC	National Collection of Type Cultures
NRRL	Agriculture Research Culture Collection
OD_630_	optical density at 630 nm
PA	*Pseudomonas aeruginosa*
PABA	*p*-aminobenzoic acid
QS	Quorum sensing
SA	*Staphylococcus aureus*
SE	*Staphylococcus epidermidis*
SM	*Streptococcus mutans*
ST	*Salmonella typhymurium*
TI	*Trichophyton* *interdigitale*
VRSA	vancomycin-resistant *Staphylococcus aureus*
